# Probabilistic motor sequence learning in a virtual reality serial reaction time task

**DOI:** 10.1371/journal.pone.0198759

**Published:** 2018-06-12

**Authors:** Florian Sense, Hedderik van Rijn

**Affiliations:** 1 Department of Experimental Psychology, University of Groningen, Groningen, The Netherlands; 2 Behavioral and Cognitive Neuroscience, University of Groningen, Groningen, The Netherlands; Anglia Ruskin University, UNITED KINGDOM

## Abstract

The serial reaction time task is widely used to study learning and memory. The task is traditionally administered by showing target positions on a computer screen and collecting responses using a button box or keyboard. By comparing response times to random or sequenced items or by using different transition probabilities, various forms of learning can be studied. However, this traditional laboratory setting limits the number of possible experimental manipulations. Here, we present a virtual reality version of the serial reaction time task and show that learning effects emerge as expected despite the novel way in which responses are collected. We also show that response times are distributed as expected. The current experiment was conducted in a blank virtual reality room to verify these basic principles. For future applications, the technology can be used to modify the virtual reality environment in any conceivable way, permitting a wide range of previously impossible experimental manipulations.

## Introduction

Nissen and Bullemer [1] introduced the serial reaction time task (SRTT) to study differences between introspective and performance measures of learning. Since their introduction of the task, it has been used widely as a way to “explore the processes underlying a broad range of behaviors, including the cognitive and biological principles of learning and memory” ([2], p. 10073). In the SRTT, learning is operationalized as a speed-up in response times (RTs) to a sequence of stimuli. Specifically, four target positions are shown horizontally on screen and are mapped onto four buttons on the keyboard. The participant simply presses the corresponding key as quickly as possible when a target lights up. A baseline for RTs can be obtained by starting the task with a block in which the targets light up randomly (excluding repetitions of the same position). Then, a repeating sequence of target positions is introduced. By using relatively long sequences (e.g., 10 items as in [1]), participants will remain unaware of a repeating pattern even though their RTs decrease (for an explicit learning variation, see, e.g., [3]). Learning measures are then derived by comparing RTs on random and sequenced blocks [2].

Roughly a decade after its introduction, Schvaneveldt and Gomez [4] proposed a probabilistic version of the SRTT. Instead of comparing performance in un-sequenced blocks with performance in sequenced blocks, the target position on any given trial has a probabilistic dependency on the target position of the previous trial. Specifically, there are two sequences, one with a high and one with a low probability. The two sequences used in Schvaneveldt and Gomez [4] are A = (1, 2, 4, 3) and B = (1, 3, 4, 2) and are recycled sequentially to generate stimulus sequences for the experiment. If A is the high-probability sequence for a given participant, for example, a target in position 1 (on screen) will be followed by either a target in position 2 (selected from sequence A) or 3 (from sequence B), with a high and low probability, respectively. Which sequence a target is drawn from on any given trial is determined by a weighted coin flip. In each case, every other target position is independent of whether A or B is the high-probability sequence (i.e., 1 and 4 are at the same location in sequence A and B). Since both A and B contain all target positions equally often, targets will, on average, appear equally often at each position. Thus, it is not that participants learn that one position is generally more frequent but that certain positions are more likely to be the target than another *given the previous target*. Hence, participants learn to anticipate certain probabilistic transitions rather than a fixed sequence.

In this context, learning of the probabilistic transitions can be assessed by contrasting responses on trials corresponding to the probable and improbable sequences. Manipulating the conditional probabilities of transitions has several advantages: Error rates become informative, especially on improbable transitions, because most incorrect responses correspond to the probable target, which indicates learning and anticipation of the probable sequence [4]. Furthermore, the learning of the probabilistic sequence can be more easily distinguished from overall on-task speed-ups in RTs. One would expect participants to get faster as a function of the number of completed trials even if there is no embedded sequence. If each trial is a probable or improbable transition, one can scrutinize the differences in RTs (or error rates) between each transition type as an interaction with trial number.

Due to its simplicity, the SRTT is a popular lab task used to study a wide range of phenomena related to learning and memory [2]. With the advance of new technology comes the opportunity to expand the set of possible experiments to run and manipulations to implement. The advent of wearable virtual reality (VR) headsets as well as the increased accessibility and usability of software to create VR environments is particularly exciting in this regard [5,6]. Rizzo and Koenig [7] surveyed the development of VR technology for clinical applications and concluded that VR is ready for primetime and has similar potential for many areas of psychology. VR also has great potential for the neuroscientific study of social processes because it can provide stimulus material that more closely resembles real world activities and interactions, and Parsons, Gaggioli, and Riva [8] argue that VR allows experimenters to maintain control while elevating ecological validity. Neguţ, Matu, Sava, and David [9] present a meta-analytic review of task difficulty when neuropsychological tests are administered either in VR or as classical pen-and-paper or computerized versions. They conclude that cognitive performance is poorer in VR, likely due to higher task complexity, which might consume additional cognitive resources. In another meta-analysis of VR measures of neuropsychological assessment, however, they show that VR measures have the necessary sensitivity to detect cognitive impairment, and suggest that VR measures have potential for many neuropsychological assessment applications [10].

If a task or test is to be implemented in VR, however, certain aspects will have to be adapted to the new environment. For the SRTT, for example, the response options are traditionally presented horizontally on the computer screen and associated with keys on the keyboard or a response box. A keyboard is usually not available in a VR environment and using the VR controller(s) is more natural than having participants hold a response box. However, VR controllers require different muscle movements, especially as the selection of alternatives is typically performed by hand- or arm-movements to move a pointer in 3D-space. Although this is a very naturalistic response, which is immediately understood and easily performed by participants, the increased complexity of these movements, and increased noise levels associated with these movements, could potentially result in reduced signal-to-noise ratios. Here, we present data from an implementation of the probabilistic SRTT in VR, using a VR controller to provide responses, in an attempt to verify that expected learning effects can be reproduced in VR. Specifically, we expected to replicate response patterns from traditional, two-dimensional implementations of the SRTT: A general reduction of RTs over time that interacts with transition probability such that high-probability transitions become increasingly faster over time compared to low-probability transitions.

## Methods

A total of 29 participants completed the experiment. Of those, 19 were female and the average age across all participants was 20.5 (range: [18; 27], SD: 2.1). Participants were recruited from the participant pool of the University of Groningen and participated for course credit. All participants gave written informed consent and the study was approved by the Ethics Committee Psychology (ID: 16300-S-NE). All students in the participant pool were eligible for participation and none were excluded based on their on-task performance. An experimental session started with the experimenter describing in detail what the participant was asked to do and a detailed explanation of the information on the informed consent forms, explaining in particular that participants can stop at any moment (without any penalty) and that they should inform the experimenter in case they are uncomfortable at any point. None of the participants indicated any discomfort or withdrew from the study. The HTC Vive accommodates most prescription glasses and we did not specify that wearing glasses would exclude participants from participating. A handful of participants did wear their glasses during the experiment but did not report any discomfort and none of the glasses were too large to cause any issues.

### Procedure

**Fig 1:**
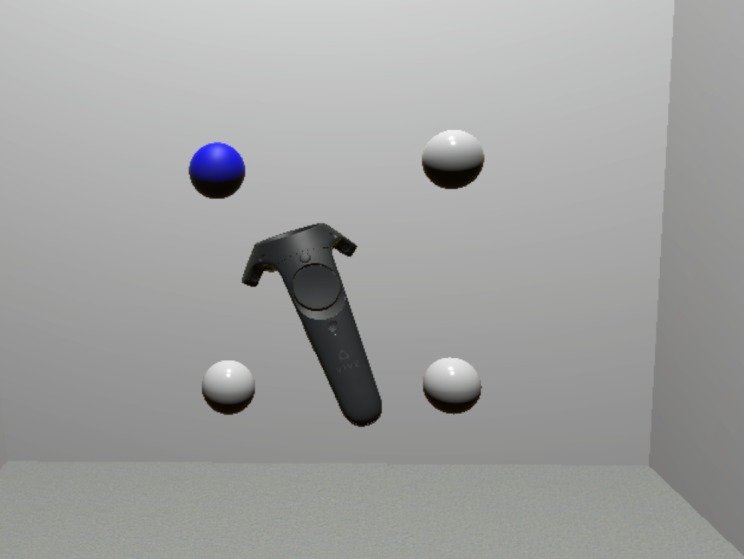
Screenshot of the VR environment. Shown is the arrangement of the four target positions while one target is lit up, indicating the participant should reach out and touch that target as quickly as possible.

Participants received general instructions for the virtual reality serial reaction time task (VR-SRTT) and then sat in a chair, wearing a HTC Vive VR headset and a single hand-held controller (in their dominant hand). The four possible target positions were presented as gray spheres in the VR environment such that they constituted the four corners of an invisible square. Participants were instructed to position themselves (and the chair they sat in) such that they could reach all four targets easily, with minimal arm movement, from a position at the center of the invisible square. A screenshot of how the environment looked like to the participant is shown in **Fig *1***.

The two sequences from Schvaneveldt and Gomez [4] were used to generate probabilistic target sequences. Which of the two served as the high probability sequence for each participant was determined randomly, and high-transition probabilities occurred in 65% of trials (compared to 80% in the original study). On each trial, the target sphere’s color changed to blue until the participant used the controller to reach out to one of the spheres. As soon as the controller intersected with any sphere, a response was recorded. Each response had two components: accuracy (correct if target sphere was touched, incorrect otherwise) and RT (the time, in milliseconds, between the lighting up of the target sphere and the moment any sphere was touched). After a response was detected, visual (corrective) feedback was provided, and the 500 ms inter-stimulus interval commenced.

First, participants completed 25 practice trials in which the order of the targets was entirely random (but target positions could not repeat). Next, they completed four blocks with 150 trials each. Every block was followed by a self-paced break to minimize fatigue. Completing the task took between 4 and 8 minutes from the moment the practice part was completed.

In the context of this project a number of distractors and their possible impact on RTs were tested. The distractors were different sounds and slight changes in the VR environment (e.g., flickering lights) and were found not to influence RTs at all. Therefore, the general learning effects reported in the Results section will be presented independently of the presence/absence of distractors. We refer the interested reader to the online supplement at https://osf.io/exnvd/, which includes a detailed description of the individual distractors, a number of annotated analyses of the possible effects of these distractors, information about the randomization procedure used for distractors, as well as the audio files used as distractors (see sub-section “Virtual Reality Serial Reaction Time Task (VR-SRTT)” in https://osf.io/vxyka/ as well as the sound files in task/stressors/ at https://osf.io/exnvd/).

## Results

Performance during the 25 practice trials was near-perfect for all participants: Overall 98.8% of responses were correct and no-one made more than two errors. This indicates that the task was understood intuitively and immediately. The data from the practice trials were discarded. Performance during the task itself was also near-perfect: Incorrect responses only accounted for 0.6% of all trials. Responses were also rather fast: The median response time (RT) across all correct responses was 425 ms and 90% of the responses were between 302 and 603 ms, with only 0.5% of all correct trials resulting in RTs longer than one second. For all subsequent analyses, we removed incorrect trials and those with RTs longer than one second. This leaves data from 17,214 trials across 29 participants.

**Fig 2:**
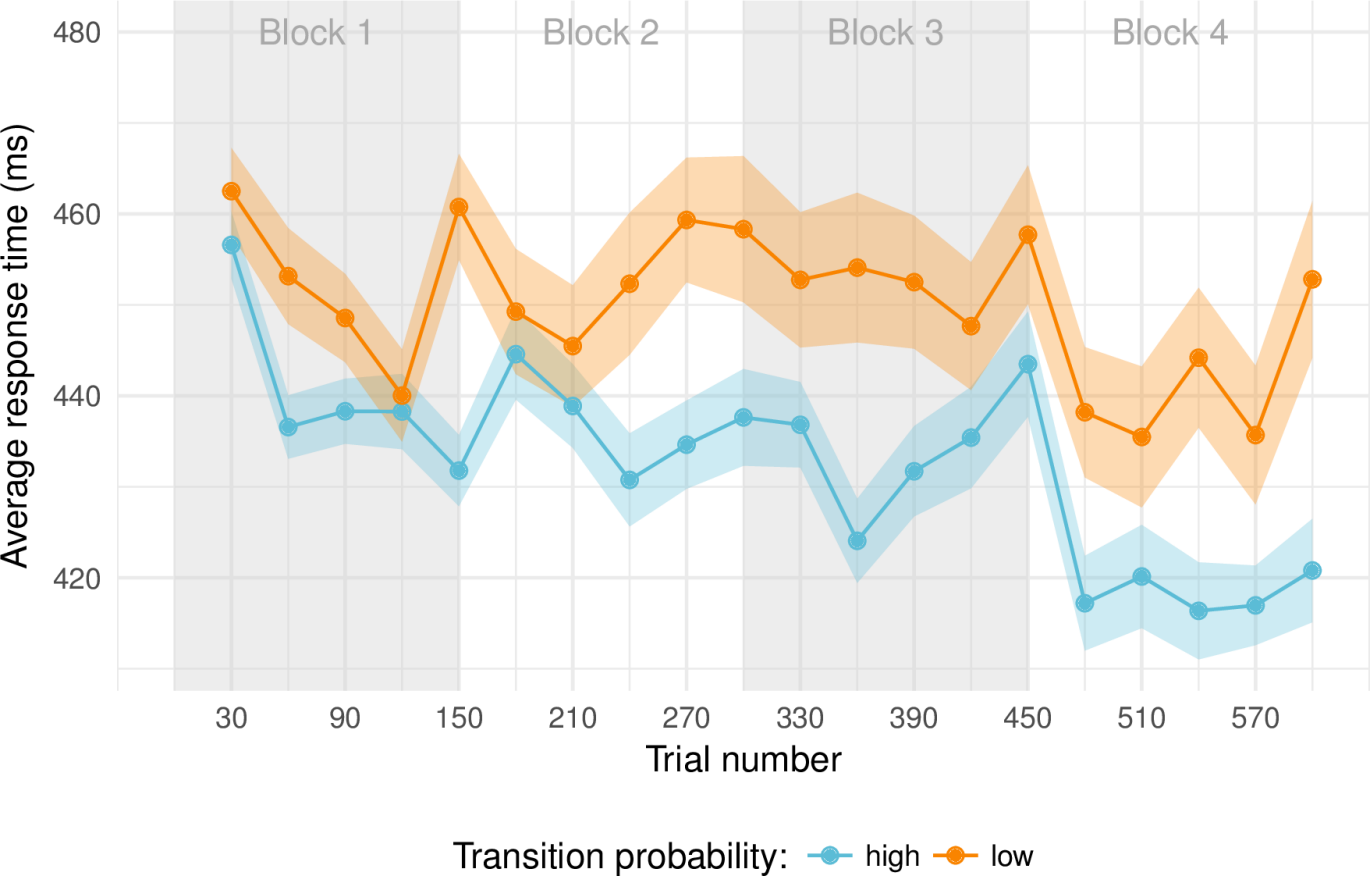
Average response time across trials. Trials have been binned to emphasize the overall pattern. Error bars are within-subject standard errors [11]. Note that there are fewer trials in the low-probability transition condition, resulting in slightly wider error bars.

We expected participants to respond faster on high-probability transitions than on low probability transitions and also expected a general speed-up as the task progresses. The aggregate data are presented in ***Fig 2*** and follow the expected pattern. Bins with 30 trials each were created and means and within-subject standard errors [11] were computed for each bin. The RTs on the high-probability transitions are consistently faster than those on the low-probability transitions. Furthermore, RTs are lower for later blocks and the difference between the two transition probability conditions increases for later blocks. The plot also highlights that differences in RTs emerge early on.

For the statistical analysis, a series of Bayesian linear mixed-effects regression models were fit to the RTs of each trial, using transition probability, trial number, and their interaction as predictors. To account for between-subject variance in RTs, random intercepts for participants were added. The models are shown in **Table *1*** along with the Bayes factors. To ease interpretation, the Bayes factors are shown relative to the worst model, revealing that, for example, the model including only transition probability as a main effect (model 2) is approximately one quadrillion times more likely to have generated the observed data than the model including only trial number as a predictor (model 1). The best-fitting model included all listed predictors (model 4) and is approximately 5 times more likely than the model including both main effects but no interaction (model 3; 1.161×10^34^ / 2.258×10^33^ = 5.14). See the supplement for the model fitting and selection procedure using Bayes factors (using the *BayesFactor* package, [12]). Also included in the supplement are estimates of the model’s coefficients as well as a traditional linear mixed-effects regression (using the *lme4* package, [13]) for comparison. See sub-section “Traditional analysis using lme4” in the file “VR-SRTT_analyses.html” at https://osf.io/exnvd/.

**Table 1:** Summary of the Bayesian linear mixed-effects regression model comparison results.

Linear Mixed-Effects Models	Bayes factors relative to 1.
1. Trial Number	1
2. Transition Probability	1.057×10^15^
3. Trial + Probability	2.258×10^33^
4. Trial + probability + Trial:Probability	1.161×10^34^

All four models include random effects for participants and Bayes factors are expressed relative to the worst-fitting model to ease interpretation.

The best-fitting model revealed by the Bayes model comparison confirms the pattern apparent in ***Fig 2***: RTs on probable transitions are estimated to be faster than those on improbable transitions and each additional trial reduces the expected RT further. However, this reduction as a function of trial number interacts significantly with transition probability such that RTs decrease more for probable transitions than for improbable transitions as trials progress.

## Discussion

The goal of the current study was to test whether probabilistic motor sequence learning effects observed in the traditional serial reaction time task (SRTT) can be reproduced in a virtual reality (VR) environment. The graphical overview in ***Fig 2*** along with the statistical analysis confirm that we see the expected interaction effects: Participants respond faster to high-probability transitions and their general speed-up over trials is more extreme for high-probability transitions.

These findings are reassuring and open a new avenue for controlled experimental studies using a VR SRTT. In the current experiment, the VR environment was a featureless gray room. One of the advantages of VR, however, is that the environment can be manipulated in any conceivable way [6]. A series of studies have demonstrated the viability and usefulness of such an approach for the Stroop task: Parsons, Courtney, and Dawson [14] have shown that traditional Stroop effects can be reproduced in VR while varying the threat-level of the VR environment (also see [15]). Parsons and Barnett [16] showed that Stroop effects still emerge when the task is presented in a VR apartment environment and are comparable to pen-and-paper and computerized Stroop tasks. Similarly, Parsons and Carlew [17] had participants complete a Stroop task in a virtual classroom and showed that individuals with autism spectrum disorder performed worse in the presence of distractors. This line of research sets promising precedents and analogous manipulations–that are much more immersive than the featureless gray room used in the present study–could be implemented for the SRTT to test their potential effects on motor sequence learning.

The current work could also be extended by recording additional measures while the task is performed. For example, Kachergis, Berends, de Kleijn, and Hommel [18] have presented analyses of mouse trajectories recorded during the performance of an SRTT. Their work could be extended into the third dimension by tracking the trajectories of the VR controller, revealing anticipation and prediction of the learned probabilistic structure of the task. Furthermore, several commercial options are available to record eye-movements in a VR headset, providing yet another way to measure anticipation, prediction, and the deployment of attention during the task.

An additional question is to which extent the responses collected in the VR environment are comparable to those collected in traditional lab settings. **Table *2*** presents RTs and error rates from the original SRTT studies by Nissen and Bullemer [1] and Schvaneveldt and Gomez [4] along with a number of more recent studies. For comparison, the overall mean RT across all 17,214 trials included in our analyses is 439ms, while it is 433ms and 450ms for the probable and improbable transition conditions, respectively, and these numbers are also listed in the table. Mean RTs from the present study seem to be in line with most of the means in **Table *2***.

**Table 2:** Comparison of reaction times across studies.

Study	Reaction Times (ms)	Error Rates (%)
Present study	Overall mean: 439 Probable: 433 Improbable: 450	Overall: 0.603 Probable: 0.450 Improbable: 0.875
Nissen & Bullemer [1]	Sequence: 216 Random: 346	Sequence: 3.250 Random: 4.625
Schvaneveldt & Gomez [4]	Probable: 368 Improbable: 447	Probable: 3.954 Improbable: 10.190
Franklin, Smallwood, Zedelius, Broadway, & Schooler [19]	Sequence: 420 Random: 436	Overall: 7.5
Du, Prashad, Schoenbrun, & Clark [20]	Overall mean: 448	N/A
Kraeutner, Gaughan, Eppler, & Boe [21]	Implicit: 610 Random: 641	Implicit: 1.55 Random: 2.75
Guzmán Muños [22]	Overall mean: 391	Overall: 4.775

All reaction times are in milliseconds (ms) and all error rates are in percent (%).

The RTs reported in the table come from a range of different populations and experimental setups so it is not surprising that there is some variance. Given that learning in the SRTT is conceptualized as a relative speed-up in RTs rather than their absolute values, however, makes it more important that our analyses confirm the presence of the expected pattern. It is reassuring, however, that the RTs collected in our experiment are not out of line with other studies, although they required a more complex motor response (moving of hand/wrist/arm rather than a button press).

Our error rates, on the other hand, are lower than those reported in any other listed study. One would expect participants to make more errors on improbably transition trials (in the probabilistic paradigm) or random blocks (in the deterministic paradigm) and this pattern is observed both in our data as well as all other studies reported in **Table 2**. The error rates column suggests that error rates vary greatly between studies but that none of them are as low as the ones reported here. The current study does not allow us to pinpoint the source of this difference. We believe that the type of motor response that is required to give a response (pressing a button vs. reaching out to a sphere) would be a prime candidate for further investigation into this difference and that recording the response trajectories through 3D space would be a promising way to illuminate this issue.

To summarize, the experiment reported here is comparable to traditional versions of the (probabilistic) serial reaction time task despite having been implemented in a novel virtual reality environment. Learning effects emerge as expected and can be traced using the change in response time distributions as in the traditional setting. These results are reassuring and open the door to innovative experimental manipulations for an established experimental paradigm using state-of-the-art technology.
